# Phorbol-12-myristate 13-acetate inhibits Nephronectin gene expression via Protein kinase C alpha and c-Jun/c-Fos transcription factors

**DOI:** 10.1038/s41598-021-00034-x

**Published:** 2021-10-13

**Authors:** Mitsuhiro Kinoshita, Atsushi Yamada, Kiyohito Sasa, Kaori Ikezaki, Tatsuo Shirota, Ryutaro Kamijo

**Affiliations:** 1 Department of Biochemistry, School of Dentistry, Showa University, 1-5-8 Hatanodai, Shinagawa-ku, Tokyo, 142-8555 Japan; 2 Department of Oral and Maxillofacial Surgery, School of Dentistry, Showa University, 2-1-1 Kitasenzoku, Ohta-ku, Tokyo, 145-8515 Japan

**Keywords:** Cell signalling, Gene regulation

## Abstract

Nephronectin (Npnt) is an extracellular matrix protein and ligand of integrin α_8_β_1_ known to promote differentiation of osteoblasts. A search for factors that regulate *Npnt* gene expression in osteoblasts revealed that phorbol 12-myristate 13-acetate (PMA), which activates protein kinase C (PKC), had a strong effect to suppress that expression. Research was then conducted to elucidate the signaling pathway responsible for regulation of *Npnt* gene expression by PMA in osteoblasts. Treatment of MC3T3-E1 cells with PMA suppressed cell differentiation and *Npnt* gene expression. Effects were noted at a low concentration of PMA, and were time- and dose-dependent. Furthermore, treatment with the PKC signal inhibitor Gö6983 inhibited down-regulation of *Npnt* expression, while transfection with small interfering RNA (siRNA) of PKCα, c-Jun, and c-Fos suppressed that down-regulation. The present results suggest regulation of *Npnt* gene expression via the PKCα and c-Jun/c-Fos pathway.

## Introduction

The extracellular matrix surrounding cells is known to be involved in various biological functions, such as cell proliferation, differentiation, and apoptosis^1–3^. Several studies have suggested that the interaction of cells with the extracellular matrix is indispensable for histogenesis and maintenance of biological functions^4, 5^. Nephronectin (Npnt) is an extracellular matrix protein considered to play critical roles in the development and function of various tissues^6, 7^. *Npnt* gene expression is seen in calcification tissues, especially in osteoblasts, thus in order to investigate osteoblast functions, we have performed experiments to elucidate the pattern of *Npnt* gene expression with several different reagents. In previous studies, we found that 1α,25-dihydroxyvitamin D_3_ and Wnt3a promoted *Npnt* gene expression^8, 9^, whereas TGF-β, TNF-α, IL-1β, OSM, FGF-2, and inorganic phosphate suppressed that expression^10–15^. Those results suggest that *Npnt* gene expression in osteoblasts is regulated via various factors. In a study conducted by Kahai et al*.*, an osteoblast-transfected *Npnt* gene expression vector was shown to promote differentiation^16^. Moreover, that differentiation was strongly promoted in cells in which the expressed region included EGF repeats. Also, in osteoblasts showing a high level of expression of mRNA 3’UTR in the *Npnt* gene, the calcification nodule was highly promoted^17^.

PMA is a phorbol ester from the spurge family of plants and the main ingredient in croton oil, which causes strong carcinogenetic promotion activity. Protein kinase C (PKC), which is activated by PMA, is a family of serine-threonine kinases that catalyze various biochemical reactions critical for the function of many cellular components, such as cell differentiation and proliferation^18, 19^. The PKC family consists of 13 isoforms that can be divided into four subgroups based on their activated pattern^20^. Classical PKCs (cPKCs; α, βI, βII, γ) require Ca^2+^/diacylglycerol (DAG)/phosphatidylserine (PS), new PKCs (nPKCs; δ, ε, η, θ) require DAG/PS, and atypical PKCs (aPKCs; λ/ι, ζ) require PS, while so-called PKC-related kinases (PRKs; 1, 2, 3), which are structurally distinct PKCs, require only PS for activation^20^. Activator protein 1 (AP-1) is a dimer consisting of the c-Jun, c-Fos, activating transcription factor (ATF), and musculoaponeurotic fibrosarcoma (MAF) families^21^. In most cells, the AP-1, a Jun/Fos heterodimer, has a high affinity for binding to the PMA response component, thus is considered to be an AP-1 site^22^. It has also been reported that tumor promoters, such as PMA and epidermal growth factor, induce AP-1 activity^23^. The relationship of PKC and AP-1 has been investigated by analyses of their molecular mechanisms^24, 25^.

In the present study, PMA was found to strongly inhibit *Npnt* gene expression through PKCα and the c-Jun/c-Fos pathway.

## Results

### PMA suppresses BMP-2 induced osteoblast differentiation in MC3T3-E1 cells

**Figure 1:**
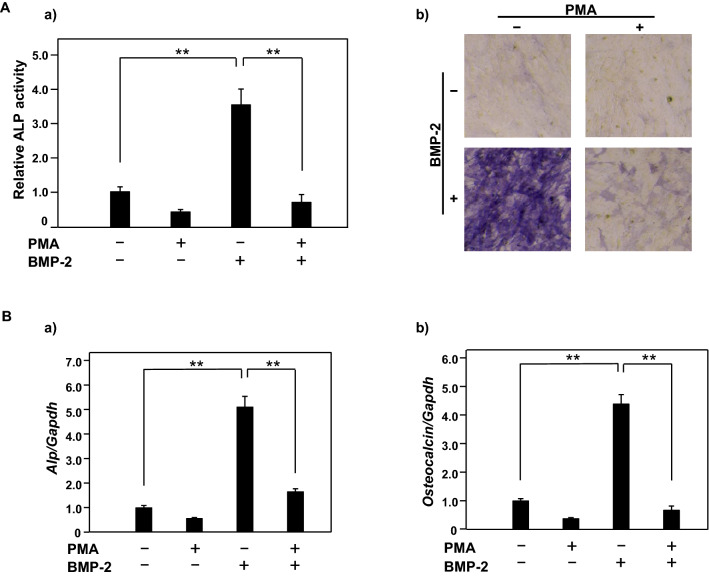
Effects of PMA on BMP-2 induced osteoblast differentiation in MT3T3-E1 cells. (**A**) (**a**) MC3T3-E1 cells were treated with or without BMP-2 (100 ng/ml) in the presence or absence of PMA (5 nM) for three days. For quantification of ALP activity, cells were disrupted by sonication in 50 mM Tris–HCl containing 0.1% NP40. ALP activity was determined following incubation with the substrate p-nitrophenylphosphate and using absorbance at 405 nm. (**b**) For ALP staining, cells were fixed using 10% formalin in PBS and then ALP activity was visualized using a mixture of 0.1 mg/ml Naphthol As-Mx, 0.6 mg/ml phosphate, and Fast blue BB salt. (**B**) Total cellular RNA was extracted, then mRNA levels of *Alp, Osteocalcin*, and *Gapdh* were examined using quantitative real-time PCR analysis. Results are shown as the mean ± SD of three samples. **P < 0.01, Student’s t test.

**Figure 2:**
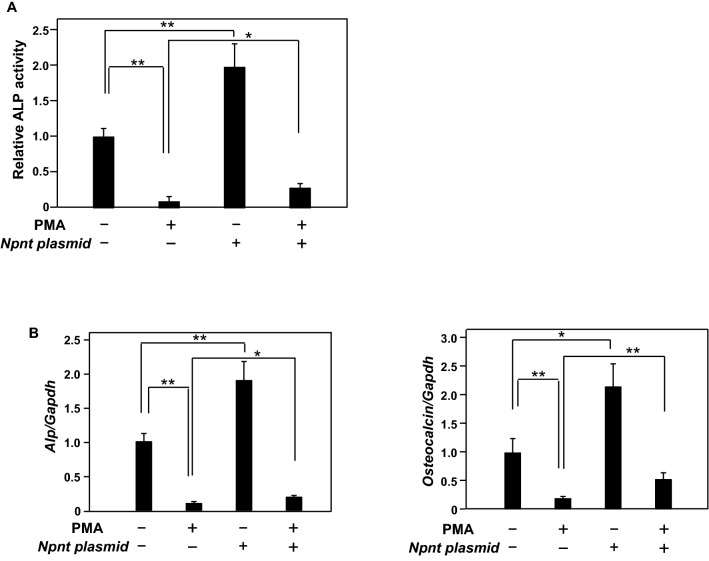
MC3T3-E1 cells with over-expression of Npnt were treated with PMA (10 nM). (**A**) For quantification of ALP activity, cells were disrupted by sonication in 50 mM Tris–HCl containing 0.1% NP40, then the activity was determined following incubation with the substrate p-nitrophenylphosphate using absorbance at 405 nm. (**B**) Total cellular RNA was extracted, then mRNA levels of *Alp, Osteocalcin*, and *Gapdh* were examined using quantitative real-time PCR analysis. Results are shown as the mean ± SD of three samples. **P < 0.01 and *P < 0.05, Student’s t test.

To investigate the effect of PMA on osteoblastic differentiation, MC3T3-E1 cells were cultured with BMP-2 (100 ng/ml) in the absence or presence of PMA (5 nM) for three days. ALP activity in cells cultured with BMP-2 was shown to be increased, whereas it was significantly suppressed when cells were cultured in the combination of BMP-2 and PMA (Fig. 1A). At the same time, the gene expressions of *Alp* and *Osteocalcin*, differentiation markers of osteoblasts, were investigated. Both *Alp* and *Osteocalcin* gene expressions induced by BMP-2 were suppressed by PMA. These results showed that PMA suppressed BMP-2 induced osteoblast differentiation (Fig. 1B)^26^. To elucidate the relationship between inhibition of osteoblast differentiation and reduction of *Npnt* gene expression by PMA, over-expression of Npnt was induced using an *Npnt* expression vector (Npnt-pCMV6-Entry) in PMA-treated MC3T3-E1 cells. *Npnt* expression reduced the level of inhibition of osteoblast differentiation by PMA (Fig. 2A,B) (Suppl. Figure 1).

### Npnt gene expression is suppressed by PMA in dose and time-dependent manner

**Figure 3:**
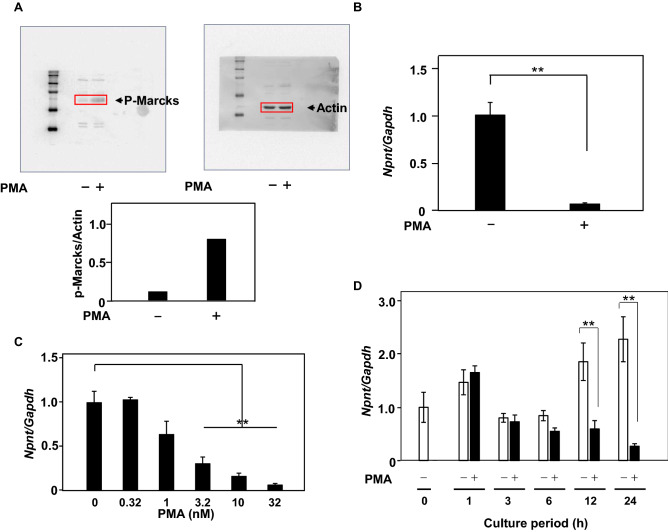
Effects of PMA on *Npnt* gene expression. (**A**) MC3T3-E1 cells were starved for 16 h in serum-free medium. Cells were treated with or without PMA (100 nM) for five minutes, then proteins were extracted and subjected to western blotting to detect phosphorylation of Marcks (p-Marcks) and actin. (**B**) MC3T3-E1 cells were treated with PMA (10 nM) for 24 h. Total cellular RNA was extracted, and mRNA levels of *Npnt* and *Gapdh* were examined using quantitative real-time PCR analysis. (**C**) Dose-dependent effects of PMA on *Npnt* expression. MC3T3-E1 cells were treated with PMA (0, 0.32, 1, 3.2, 10, or 32 nM) for 24 h and then examined using quantitative real-time PCR analysis. (**D**) Time course analysis of PMA effects on *Npnt* gene expression. MC3T3-E1 cells were treated with PMA (10 nM) for 0, 1, 3, 6, 12, or 24 h and then examined using quantitative real-time PCR analysis. Results are shown as the mean ± SD of 3 samples. **P < 0.01, Student’s t-test as compared to the level with 0 nM of PMA.

PMA, a phorbol ester, is known to activate the PKC signaling pathway. To determine whether PMA activated the PKC signaling pathway in MC3T3-E1 cells, Marcks phosphorylation was examined, as previous studies have reported that it was phosphorylated by PKC activation^27, 28^ (Fig. 3A). The effect of PMA on *Npnt* gene expression was also examined and the results showed that expression to be significantly down-regulated by PMA (Fig. 3B). Next, the effects of PMA on dose- and time-dependent *Npnt* gene expression were investigated. That expression was significantly decreased by PMA at 3.2 nM and reached a plateau at 32 nM (Fig. 3C), while it was also significantly decreased by 10 nM of PMA at 12 h and then reached a plateau at 24 h (Fig. 3D). These results suggest that *Npnt* gene expression is suppressed by PMA in a dose and time-dependent manner.

### PKCα is involved in down-regulation of Npnt gene expression by PMA

**Figure 4:**
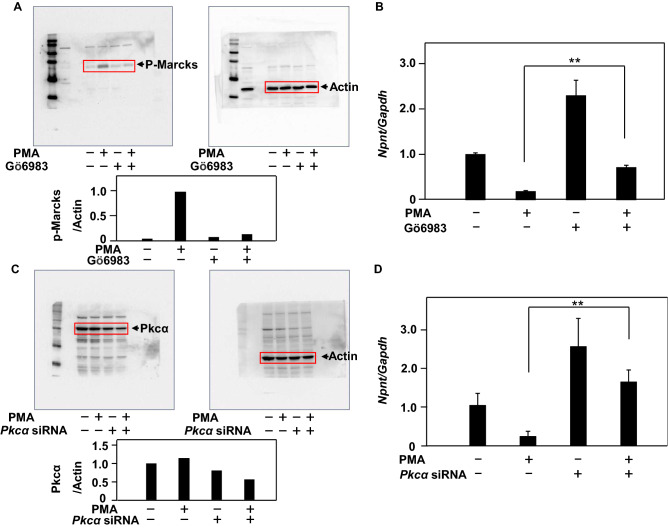
PKC signaling, especially PKCα, is involved in *Npnt* gene down-regulation by PMA. (**A**) MC3T3-E1 cells were starved for 16 h in serum-free medium. Next, they were pretreated with or without Gö6983 (500 nM) for one hour, and then with PMA (5 nM) alone or in combination for five minutes. Proteins were extracted and subjected to western blotting to detect phosphorylation of Marcks (p-Marcks) and actin. (**B**) MC3T3-E1 cells were pretreated with or without Gö6983 (500 nM) for one hour, and then treated with PMA (5 nM) alone or in combination for 24 h. Total cellular RNA was extracted, and mRNAs for *Npnt* and *Gapdh* were examined using real-time PCR analysis. (**C**) MC3T3-E1 cells were pretreated with or without *Pkcα* siRNA (20 nM) for 24 h, and then treated with PMA (10 nM) alone or in combination for 24 h. Proteins were extracted and subjected to western blotting to detect Pkcα and actin. (**D**) Total cellular RNA was extracted, and mRNAs for *Npnt* and *Gapdh* were examined using real-time PCR analysis. Results are shown as the mean ± SD of three samples. **P < 0.01, Student’s t-test, as compared to presence or absence of PMA, Gö6983, and *Pkcα* siRNA.

To verify whether down-regulation of *Npnt* gene expression by PMA is involved in the PKC signaling pathway, MC3T3-E1 cells were pretreated with Gö6983, known as a broad-spectrum PKC inhibitor, before PMA stimulation. Phosphorylation of Marks by PMA did not occur following pretreatment with Gö6983 (Fig. 4A), while down-regulation of *Npnt* gene expression by PMA was inhibited by Gö6983 (Fig. 4B). These results suggest that *Npnt* gene expression is involved in the PKC signaling pathway.

It has been reported that PKCα is highly expressed in MC3T3-E1 cells^29^. To verify its involvement in down-regulation of *Npnt* gene expression, MC3T3-E1 cells were pretreated with or without *Pkcα* siRNA, and thereafter with PMA alone or in combination. When *Pkcα* siRNA decreased the cellular protein level of Pkcα (Fig. 4C), down-regulation of *Npnt* gene expression by PMA was inhibited (Fig. 4D). These results indicate that PKCα is involved in down-regulation of *Npnt* gene expression by PMA.

### Both of c-Jun and c-Fos are involved in down-regulation of Npnt gene expression

**Figure 5:**
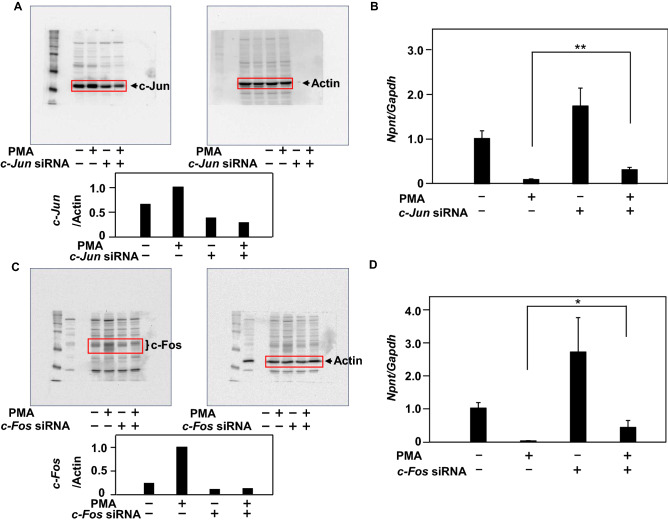
*Npnt* gene down-regulation by PMA regulated via c-Jun and c-Fos transcription factors. MC3T3-E1 cells were pretreated with or without *c-Jun* siRNA (20 nM) or *c-Fos* siRNA (20 nM) for 24 h, and then treated with PMA (100 nM) alone or in combination for 24 h with *c-Jun* or for three hours with *c-Fos*. (**A**) Proteins were extracted and subjected to western blotting to detect c-Jun and actin. (**B**) Total cellular RNA was extracted, and mRNAs for *Npnt* and *Gapdh* were examined using real-time PCR analysis. (**C**) Proteins were extracted using the same procedures shown in (**A**) and (**B**), and subjected to western blotting to detect c-Fos and Actin. (**D**) Total cellular RNA was extracted, and mRNAs for *Npnt* and *Gapdh* were examined using real-time PCR analysis. Results are shown as the mean ± SD of three samples. *P < 0.05, **P < 0.01, Student’s t-test, as compared to presence or absence of PMA, *c-Jun* siRNA, and *c-Fos* siRNA.

It has been reported that regulation of gene expression by PMA is involved in activation of PKCα and thereafter of AP-1^30^. Down-regulation of *PKCα* gene expression in MC3T3-E1 cells resulted in reduced phosphorylations of c-Jun and c-Fos (Suppl. Figure 2A,B). To investigate the involvement of c-Jun and c-Fos as transcription factors, which compose AP-1, on down-regulation of *Npnt* gene expression, MC3T3-E1 cells were pretreated with or without c-*Jun*, *c-Fos* siRNA, and then treated with PMA alone or in combination. When *c-Jun* siRNA decreased the cellular protein level of c-Jun (Fig. 5A), down-regulation of *Npnt* gene expression by PMA was inhibited (Fig. 5B), and when *c-Fos* siRNA decreased the level of c-Fos (Fig. 5C), down-regulation of *Npnt* gene expression by PMA was inhibited (Fig. 5D). These results demonstrated that the transcription factors c-Jun and c-Fos are involved in down-regulation of *Npnt* gene expression by PMA.

## Discussion

The present findings indicate that PMA, known to suppress osteoblast differentiation, downregulates *Npnt* gene expression. That downregulation was shown to be mediated via PKCα, and further via c-Jun and c-Fos, which are transcription factors in PKC signaling. Nakura et al*.*, demonstrated that knockdown of *PKCα* gene expression promoted osteoblast differentiation and their results also suggest that PKCα suppresses osteoblast differentiation^31^. Furthermore, Galea et al*.* reported that PKCα knockout mice, which show a phenotype similar to human Gaucher disease, had bone formation into the medullary space of the femur. Moreover, osteoblasts derived from those mice showed elevated osteoblast differentiation markers, such as Runx2, Osterix, Col1A1, and Osteocalcin^32^. Together, these results suggest that PKCα negatively regulates bone formation. Regarding the activation of PKCα in relation to inhibition of osteoblast differentiation, Bordin et al*.* examined the physiological effect of PKCα activation and presented findings suggesting that IL-6 expression in osteoblasts was mediated by that activation, while Grano et al*.* reported that IL-6 reduced osteoblast differentiation and increased bone resorption^33, 34^. Based on those results, it is considered that IL-6, an inflammatory cytokine, suppresses osteoblast differentiation via activation of PKCα. Additionally, the present results indicate that PKCα negatively regulates promotion of osteoblast differentiation, with one of the causes considered to be a decrease in *Npnt* gene expression due to PKCα, though further studies are required to confirm that association.

c-*Jun*, *c-Fos* siRNA decreased the level of c-*Jun*, *c-Fos* mRNA, which resulted in partial recovery of down-regulation of *Npnt* gene expression by PMA. This suggests the presence of another pathway in addition to the c-Jun and c-Fos pathways for suppressing *Npnt* gene expression by PMA. Bedini et al*.*, reported that PMA treatment suppressed *hMOR* gene expression in SH-SY5Y cells, the neuroblastoma cell line. In addition, in the present study, suppression of expression of REST (repressor element 1 silencing transcription factor), a transcription factor known to be involved in regulation of gene expression in differentiated and post-differentiated neurons, inhibited PMA-induced *hMOR* gene downregulation. The hMOR promoter has been shown to have a REST binding region^35^. Furthermore, Kuan et al., reported that PMA treatment suppressed *ckβ* gene expression in MCF-7 cells, while it also suppressed the promoter activity of the *ckβ* gene^36^. That study also noted that the promoter region of the *ckβ* gene has binding sites for the transcription factors GATA and Ets, and mutations in those binding sites inhibited suppression of the promoter activity of the *ckβ* gene by PMA. Sun et al*.* also found that *Npnt* gene expression was down-regulated by TGF-β and oncostatin M in osteoblasts via MAPK signaling pathways^37^. Also, PMA is known to stimulate the MAPK pathway, while crosstalk between PKCα and MAPK signaling pathways regulates *Npnt* gene expression, though additional research is needed to verify their relationship^38^.

**Figure 6:**
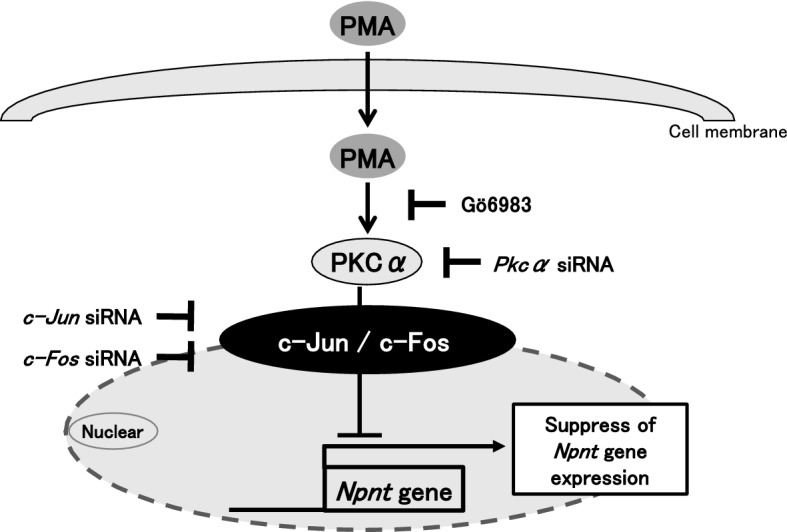
Model of down-regulation of *Npnt* gene expression by PMA. Activation of PKC signaling by PMA, *Npnt* gene expression was suppressed via the transcription factors c-Jun and c-Fos. (This image was drawn using Microsoft Office Power Point version 2018).

In conclusion, we found that PKCα suppresses *Npnt* gene expression via c-Jun and c-Fos transcription factors (Fig. 6).

## Methods

### Cell culture

The osteoblast-like cell line MC3T3-E1 was maintained in MEMα with L-glutamine and phenol red medium (FujiFilm Wako Pure Chemical Industries, Ltd., Cat. No. 135–15,175), supplemented with 10% fetal bovine serum (FBS) (Biosera, Cat. No. FB-1285) and 1% penicillin–streptomycin (Gibco, Cat.No. 15240–062) at 37˚C in a CO_2_ incubator (5% CO_2_, 95% air). Osteoblast differentiation was induced by MEMα supplemented with 10% FBS and 100 ng/ml of BMP-2 (R&D Systems, Cat. No.355-BEC-010) for three days.

### Reagents

PMA (phorbol 12-myristate 13-acetate) was purchased from Adipo Gen Life Sciences, Inc. (Cat. No. AG-CN2-0010-M001). BMP-2 human recombinant protein was purchased from R&D Systems, Inc. (Cat. No.355-BEC-010) and Gö6983 from Cayman Chemical, Inc. (Cat. No.13311). pCMV6-Entry (Cat. No. PS100001) and Npnt-pCMV6-Entry (Cat. No. MR208888) vectors were purchased from ORIGENE.

### Quantitative real-time PCR

Total RNA was extracted from cells using TRIzol® Reagent (Life Technologies, Cat. No. 15596018), then cDNA was synthesized using ReverTra Ace® qPCR RT Master Mix (TOYOBO CO., LTD, Cat. No. FSQ-201). Quantitative real-time PCR was performed using Power Up™ SYBR™ Green Master Mix (Applied Biosystems, Cat. No. A25742) or THUNDERBIRD® Probe qPCR Mix (TOYOBO CO., LTD, Cat. No. QPS-101). As another procedure, using TaqMan™ Fast Advanced Cells-to-CT™ Kit (Invitrogen) in accordance with the manufacturer’s protocol, after cells were lysed cDNA was synthesized and then quantitative real-time PCR was performed. The TaqMan™ IDs (Applied Biosystems) of the gene expression assay were as follows: *Gapdh* (Mm99999915_g1), *Alp* (Mm00475834_m1), and *Osteocalcin* (Mm03413826_mH). Following are the sequences of the specific PCR primers (Life Technologies): *Gapdh*: 5ʹ-AAATGGTGAAGGTCGGTGG-3ʹ and 5ʹ-TGAAGGGGTCGTTGATGG-3ʹ, *Npnt*: 5ʹ-CACGAGTAATTACGGTTGACAACAG-3ʹ and 5ʹ-CTGCCGTGGAATGAACACAT-3ʹ.

### Western blotting

Cells were lysed with Sample Buffer Solution with Reducing Reagent (6x) for SDS-PAGE (NAKALAI TESQUE, Inc. Cat. No. 09499–14), then the lysates were subjected to SDS-PAGE. Following electrophoresis, proteins were transferred to PVDF membranes (Merck Millipore Ltd. Cat. No. IPVH00010). The membranes were treated with specific primary antibodies reacting to phospho-Marcks, Pkcα, c-Jun, and c-Fos (Cell Signaling TCCHNOLOGY, Cat. No. 2741, 2056, 9165 and 4384, respectively), and actin (SIGMA-ALDRICH, Cat. No. A5060), followed by incubation with ECL™ Anti-Rabbit IgG and treatment with a horseradish peroxidase linked whole antibody (GE Healthcare UK Limited Cat. No. NA934V). Immuno-reactive bands were visualized using ECL™ Prime Western Blotting Detection Regents (GE Healthcare. Cat. No. RPN2232) and the intensity of chemi-luminescent bands was quantitated with Versa Doc 5000MP (Bio-Rad Laboratories, Inc.).

### ALP staining and activity

Cells were fixed with 10% formalin in PBS, then ALP activity was visualized using a mixture of 0.1 mg/ml Naphthol As-Mx (SIGMA, Cat. No. N4875), 0.6 mg/ml phosphate, and Fast blue BB salt (SIGMA, Cat. No. F3378). For quantification of ALP activity, cells were disrupted by sonication in 50 mM Tris–HCl containing 0.1% NP40 (Wako Pure Chemical Industries, Ltd., Cat. No.198596). ALP activity was determined following incubation with p-nitrophenylphosphate substrate (FujiFilm Wako Pure Chemical Industries, Ltd., Cat. No.149–02342).

### Knockdown of genes with RNA interference

Cells were transfected with Stealth™ siRNAs for mouse *Pkcα, c-Jun* siRNA, or a negative control (Invitrogen), or Silencer™ Select pre-designed siRNA for mouse c-*Fos* or a negative control (Ambion) using lipofectamine IMAX (Thermo Fisher) (Cat. No.13311), in accordance with the protocols of the manufacturers.

The respective oligos were as follows: *Pkcα*: 5ʹ-UCCAAAUGGGCUUUCGGAUCCUUAU-3ʹ and 5ʹ-AUAAGGAUCCGAAAGCCCAUUUGGA-3ʹ, *c-Jun*: 5ʹ-GAGAGCGGUGCCUACGGCUACAGUA-3ʹ and 5ʹ-UACUGUAGCCGUAGGCACCGCUCUC-3ʹ, and *c-Fos*: 5ʹ-CUACUUACACGUCUUCCUUtt-3ʹ and 5ʹ-AAGGAAGACGUGUAAGUAGtg-3ʹ.

### Statistical analysis

Values are expressed as the mean ± SD. A two-sided unpaired Student’s test was used for statistical analysis. Statistical differences were considered to be significant when the *P* value was < 0.05.

## Supplementary Information

Supplementary Information 1.

Supplementary Information 2.
