# Supplementary material for: Phorbol-12-myristate 13-acetate inhibits Nephronectin gene expression via Protein kinase C alpha and c-Jun/c-Fos transcription factors
Source: Sci Rep. 2021 Oct 13;11:20360. doi: 10.1038/s41598-021-00034-x (PMC8514542; doi:10.1038/s41598-021-00034-x)
Supplement: Supplementary file 1 — Supplementary Information 1. [file 41598_2021_34_MOESM1_ESM.pdf]

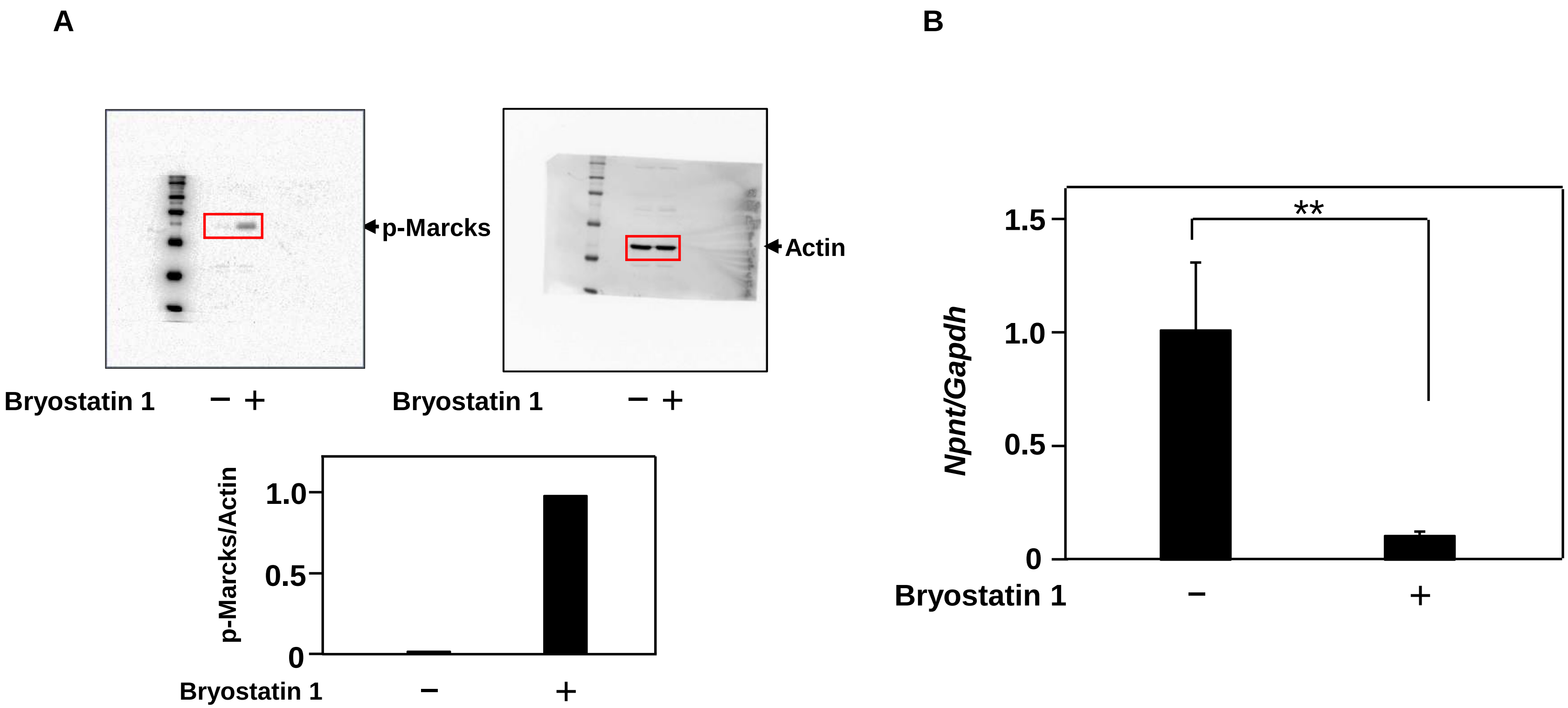

(A) Activation of PKC by Bryostatin 1. MC3T3-E1 cells were starved for 16 hours in serum-free medium. Cells were treated with or without Bryostatin (10 nM) for five minutes, then proteins were extracted and subjected to Western blotting to detect phosphorylation of Marcks (p-Marcks) and Actin.

(B) Effects of Bryostatin 1 on *Npnt* gene expression.

MC3T3-E1 cells were treated with Bryostatin (10 nM) for 24 hours. Total cellular RNA was extracted, and mRNA levels of *Npnt* and *Gapdh* were examined using quantitative real-time PCR analysis. Results are shown as the mean ± SD of 3 samples. **P <0.01, Student's t-test, as compared to presence or absence of Bryostatin 1.

Appendix Fig.1. Kinoshita, M. *et al.*

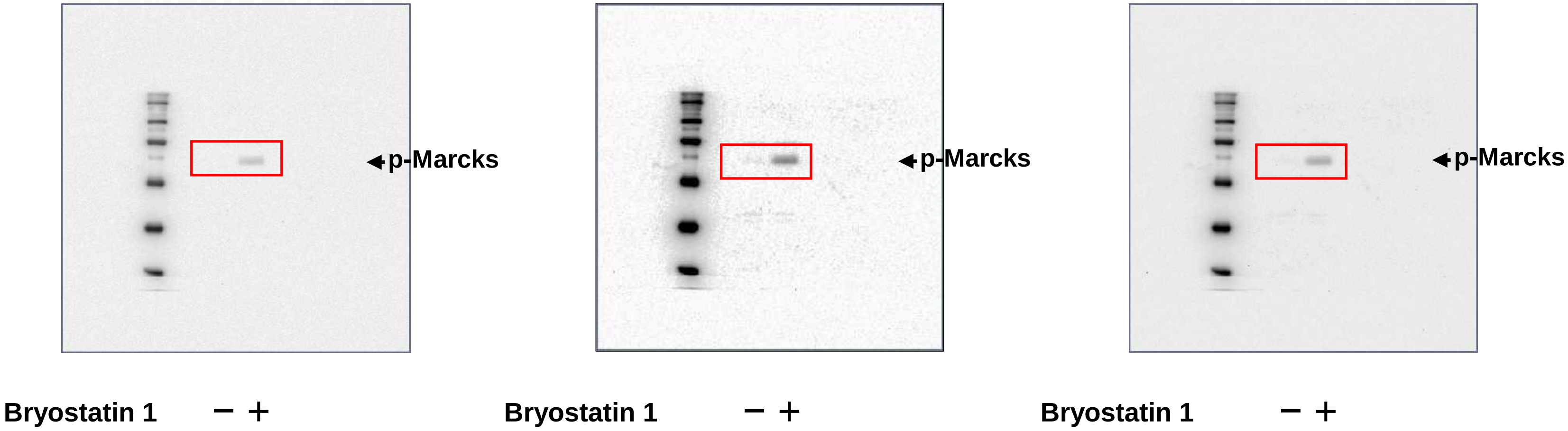

Appendix Figure 1-1. Multiple exposure images of Appendix Figure 1.

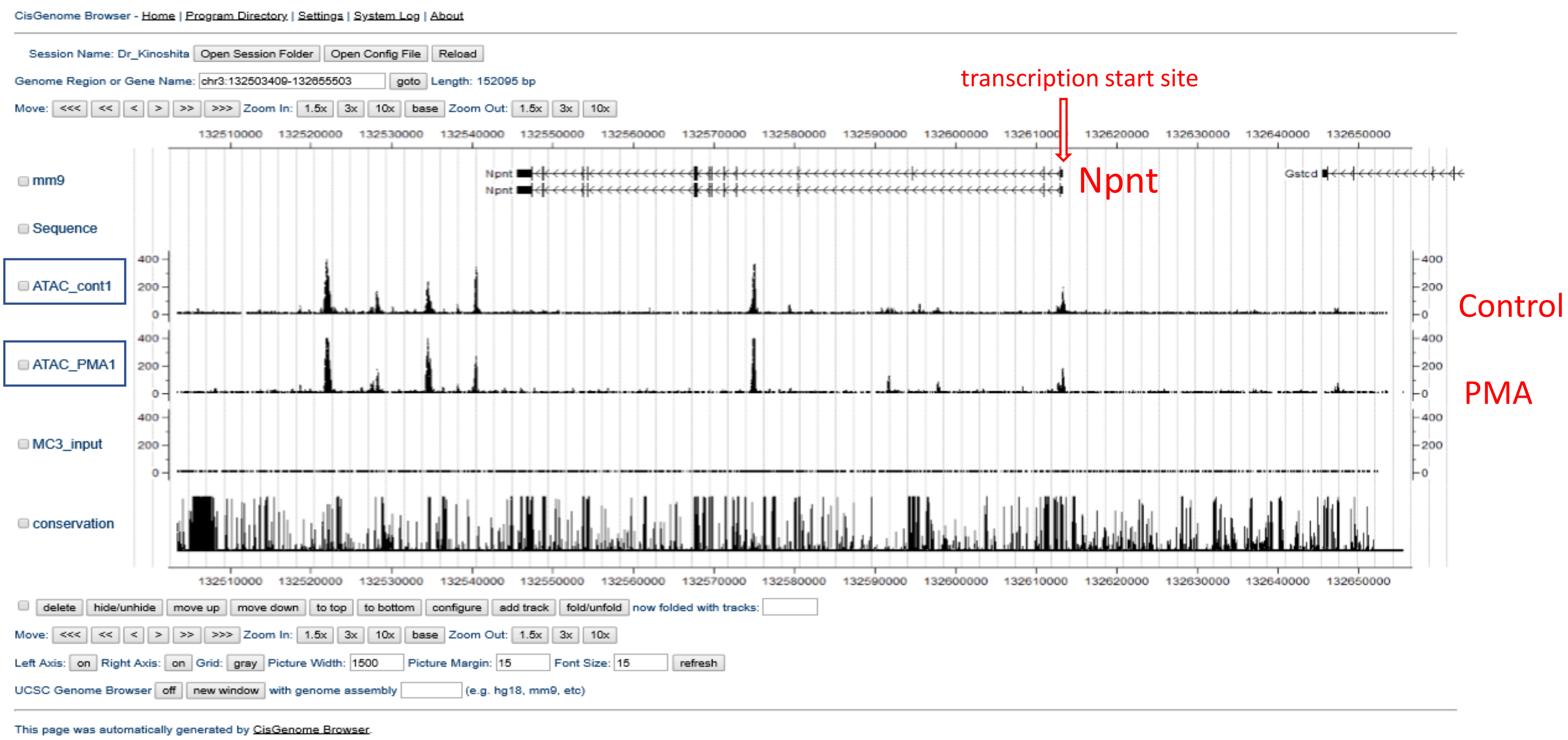

The ATAC-seq was performed as described previously [*Buenrostro JD et al., Nat Methods. 2013 Dec; 10(12):1213-8.*]. Briefly, MC3T3-E1 cells were treated in the absence or presence of PMA (100nM) for 24 hours, and then 50,000 cells were collected and lysed with lysis buffer containing 10 mM Tris–HCl, 10 mM NaCl, 3 mM MgCl2, and 0.1% IGEPAL CA-630. Tn5 transposase reaction using the Tagment DNA Enzyme 1 (TDE1) (Illumina) was carried out at 37 ° C for 30 min. The reacted DNA was purified using QIAGEN MinElute PCR purification kit and amplified for 8–15 cycles to produce libraries for sequencing. The ATAC-seq libraries were sequenced on Illumina Hiseq X sequencer. The sequence reads were aligned to the mouse genome reference sequence mm9 by bowtie aligner [*Langmead B et al., Genome Biol. 2009; 10(3):R25.*]. Peak calling was performed by two-sample analysis on CisGenome software [*Ji H et al., Nat Biotechnol. 2008 Nov; 26(11):1293-300.*].

Appendix Fig.2. Kinoshita, M. *et al.*
