# Supplementary material for: Phorbol-12-myristate 13-acetate inhibits Nephronectin gene expression via Protein kinase C alpha and c-Jun/c-Fos transcription factors
Source: Sci Rep. 2021 Oct 13;11:20360. doi: 10.1038/s41598-021-00034-x (PMC8514542; doi:10.1038/s41598-021-00034-x)
Supplement: Supplementary file 2 — Supplementary Information 2. [file 41598_2021_34_MOESM2_ESM.pdf]

Phorbol-12-myristate 13-acetate inhibits Nephronectin gene expression via Protein kinase C alpha and cJun/c-Fos transcription factors

Mitsuhiro Kinoshita[1], Atsushi Yamada[1, *], Kiyohito Sasa[1], Kaori Ikezaki[1, 2], Tatsuo Shirota[2], Ryutaro Kamijo[1]

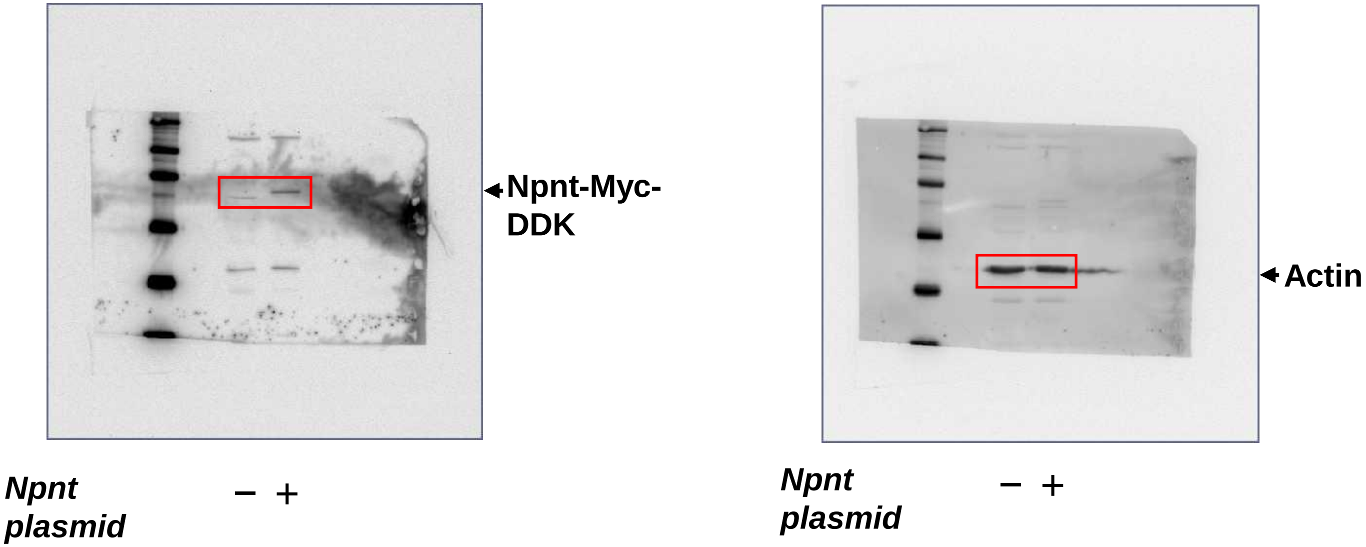

Supplementary Figure 1. Npnt over-expression in MC3T3-E1 cells was performed using Npnt in pCMV6-Entry (Npnt) and Mock (pCMV6-Entry) vectors. The PCMV6-Entry vector is a mammalian vector with a C-terminal Myc-DDK Tag. Proteins were extracted and subjected to western blotting detection of Flag (DDK) and actin [antibodies: FLAG M2 and actin (SIGMA-ALDRICH, Cat. No. A5060, F1804 respectively)].

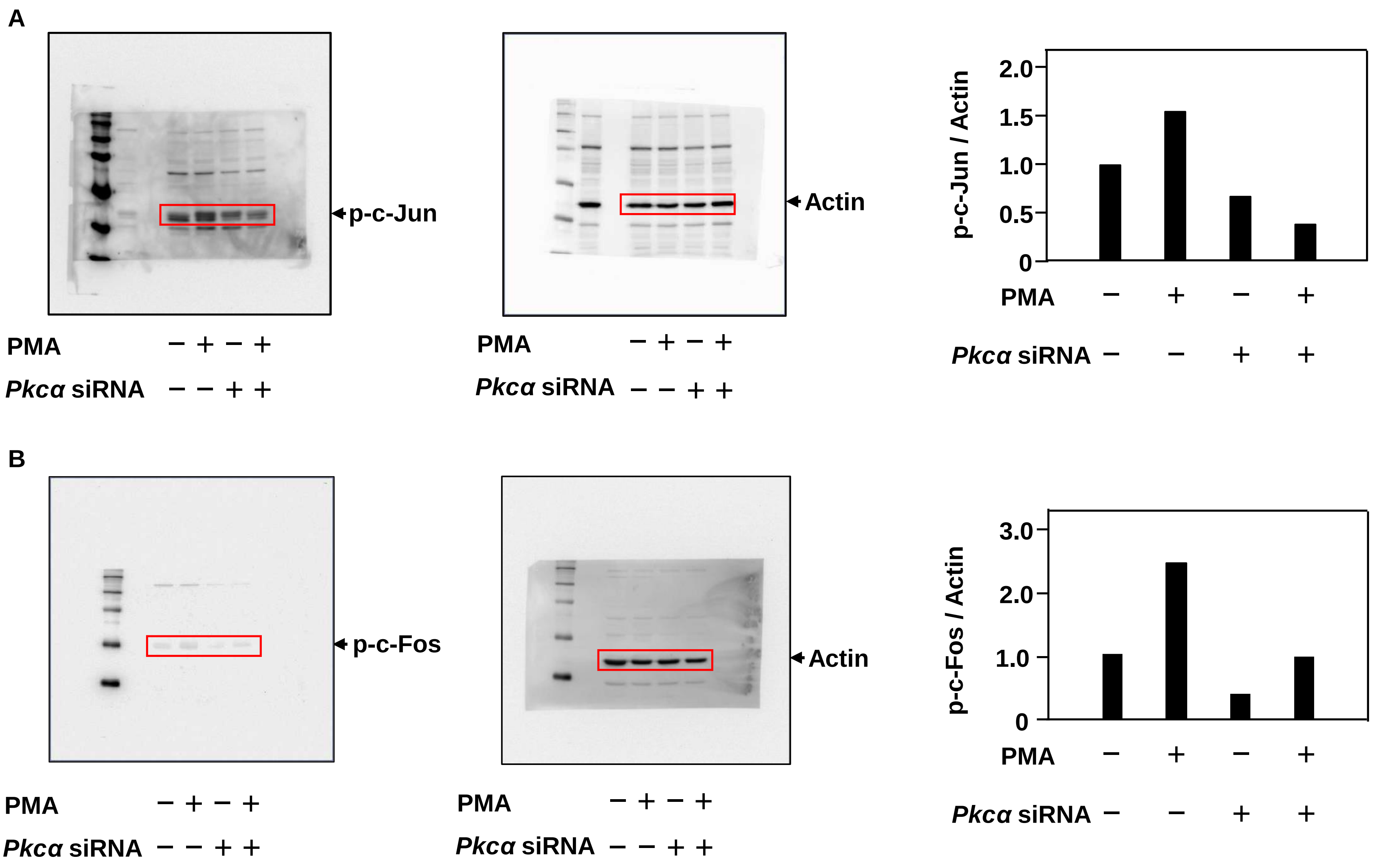

Supplementary Figure 2. Decreased expression of Pkcα suppressed phosphorylation of c-Jun and c-Fos. MC3T3-E1 cells were pretreated with or without *Pkcα* siRNA (20 nM) for 24 hours, then treated with PMA (10 nM) alone or that in combination for 24 hours. Proteins were extracted and subjected to western blotting to detect (A) p (phosphorylated)-c-Jun, and (B) p-c-Fos [antibodies: phospho-c-Jun, phospho-c-Fos (Cell Signaling TECHNOLOGY, Cat. No.3270 and 5348 respectively),].

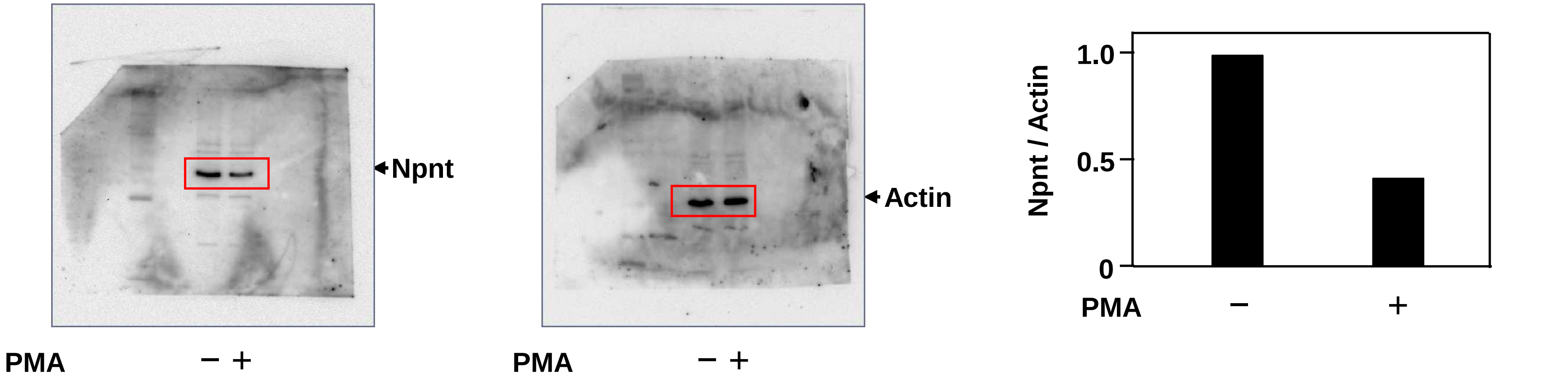

Supplementary Figure 3. Effects of PMA on Npnt protein expression. MC3T3-E1 cells were starved for 16 hours in serum-free medium. Cells were treated with or without PMA (10 nM) for 24 hours, then proteins were extracted and subjected to western blotting to detect Npnt and actin [antibodies: Npnt (SIGMA-ALDRICH, Cat. No.AV47815 and F1804 respectively)].

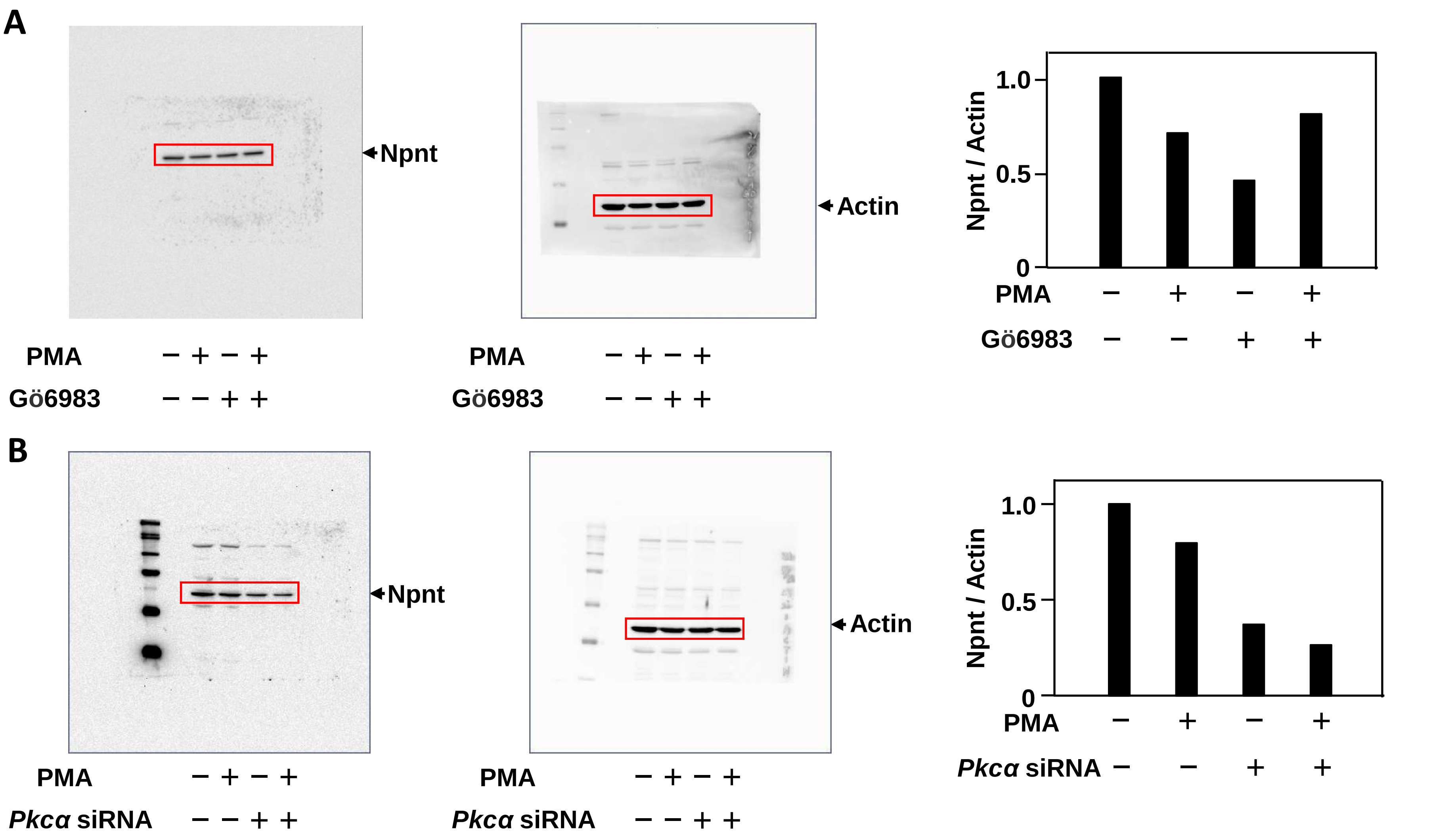

Supplementary Figure 4. Involvement of PKC signaling, especially PKCα, in down-regulation of Npnt protein by PMA. (A) MC3T3-E1 cells were starved for 16 hours in serum-free medium. Next, they were pretreated with or without Gö6983 (500 nM) for one hour, then treated with PMA (5 nM) alone or that in combination for 24 hours. Proteins were extracted and subjected to western blotting to detect Npnt and actin. (B) MC3T3-E1 cells were pretreated with or without *Pkcα* siRNA (20 nM) for 24 hours, then treated with PMA (10 nM) alone or that in combination for 24 hours. Proteins were extracted and subjected to western blotting to detect Npnt and actin [antibodies: Npnt (SIGMA-ALDRICH, Cat. No.AV47815 and F1804 respectively)].

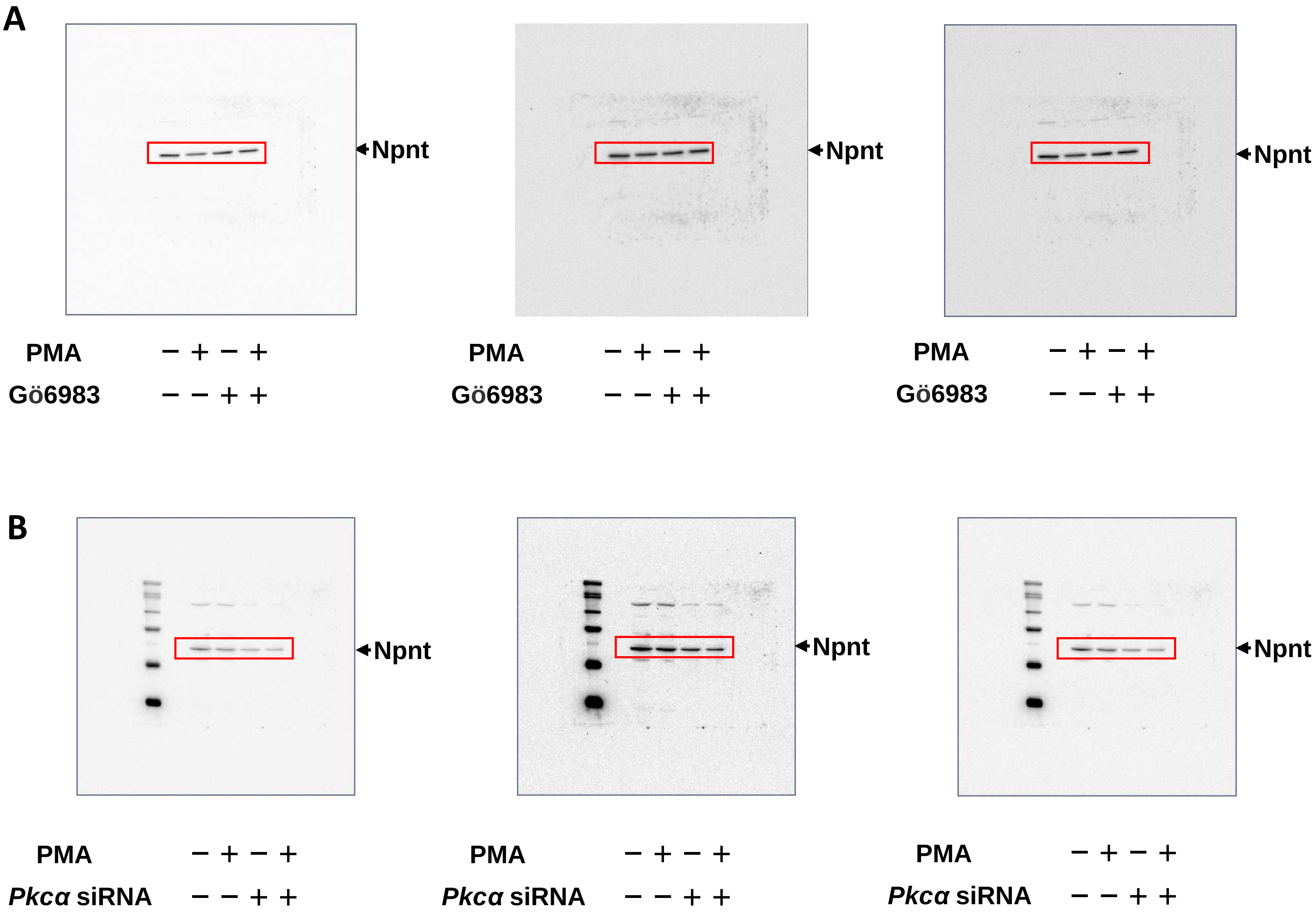

Supplementary Figure 4-1. Multiple exposure images of Supplementary Figure 4.

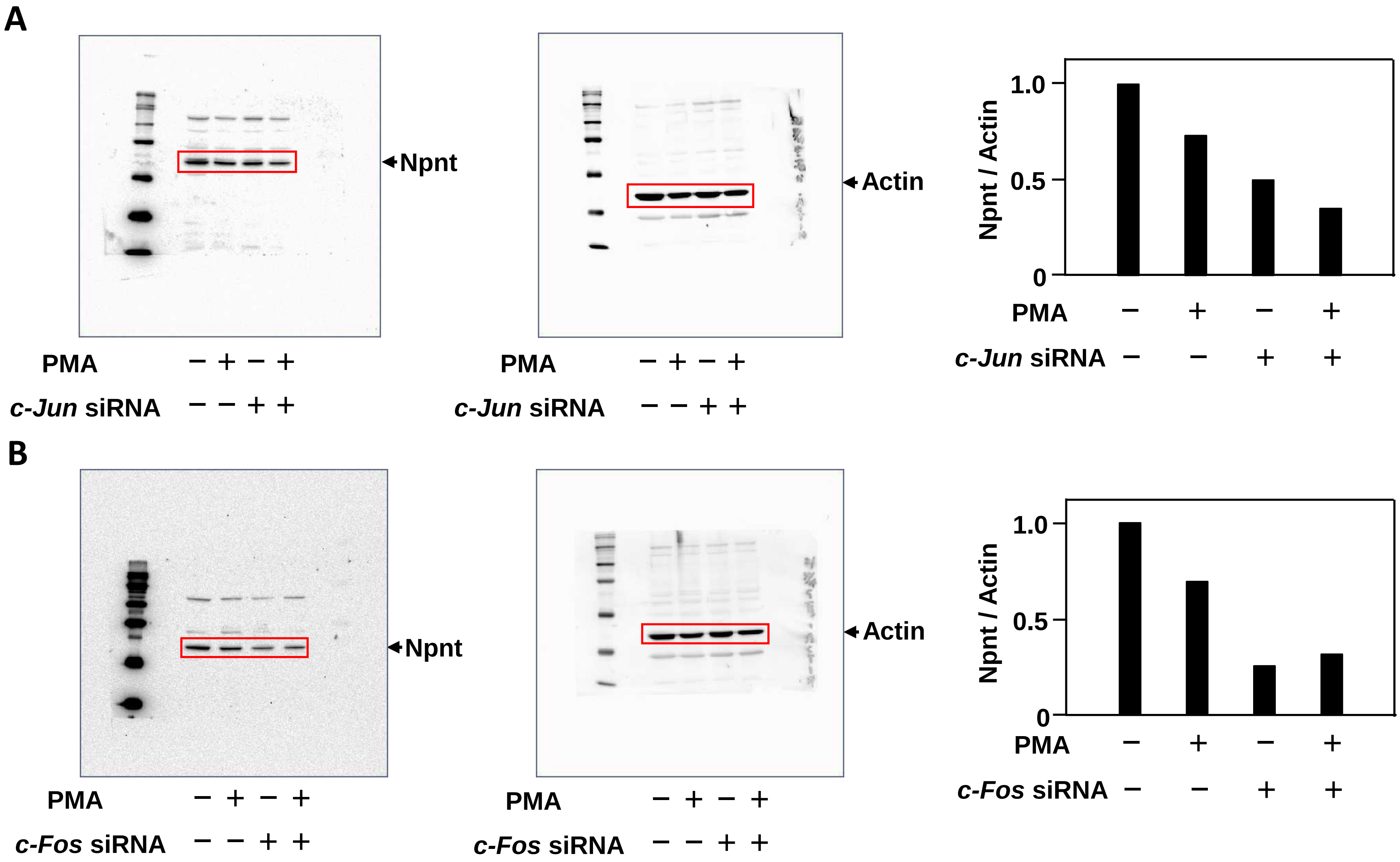

Supplementary Figure 5. Npnt protein down-regulation by PMA regulated via c-Jun and c-Fos transcription factors. MC3T3-E1 cells were pretreated with or without (A) *c-Jun* siRNA (20 nM) or (B) *c-Fos* siRNA (20 nM) for 24 hours, and then treated with PMA (100 nM) alone or in combination for 24 hours. Proteins were extracted and subjected to western blotting to detect Npnt and actin [antibodies: Npnt (SIGMA-ALDRICH, Cat. No.AV47815 and F1804 respectively)].
